# In the Absence of Frazzled Over-Expression of Abelson Tyrosine Kinase Disrupts Commissure Formation and Causes Axons to Leave the Embryonic CNS

**DOI:** 10.1371/journal.pone.0009822

**Published:** 2010-03-23

**Authors:** Joy N. Dorsten, Bridget E. Varughese, Stephanie Karmo, Mark A. Seeger, Mark F. A. VanBerkum

**Affiliations:** 1 Department of Biological Sciences, Wayne State University, Detroit, Michigan, United States of America; 2 Department of Molecular Genetics and Center for Molecular Neurobiology, Ohio State University, Columbus, Ohio, United States of America; Harvard University, United States of America

## Abstract

**Background:**

In the *Drosophila* embryonic nerve cord, the formation of commissures require both the chemoattractive Netrin receptor Frazzled (Fra) and the Abelson (Abl) cytoplasmic tyrosine kinase. Abl binds to the cytoplasmic domain of Fra and loss-of-function mutations in *abl* enhance *fra*-dependent commissural defects. To further test Abl's role in attractive signaling, we over-expressed Abl in Fra mutants anticipating rescue of commissures.

**Methodology/Principal Findings:**

The Gal4-UAS system was used to pan-neurally over-express Abl in homozygous *fra* embryos. Surprisingly, this led to a significant decrease in both posterior and anterior commissure formation and induced some commissural and longitudinal axons to project beyond the CNS/PNS border. Re-expressing wild-type Fra, or Fra mutants with a P-motif deleted, revert both commissural and exiting phenotypes, indicating that Fra is required but not a specific P-motif. This is supported by S2 cell experiments demonstrating that Abl binds to Fra independent of any specific P-motif and that Fra continues to be phosphorylated when individual P-motifs are removed. Decreasing midline repulsion by reducing Robo signaling had no effect on the Abl phenotype and the phenotypes still occur in a *Netrin* mutant. Pan-neural over-expression of activated Rac or Cdc42 in a *fra* mutant also induced a significant loss in commissures, but axons did not exit the CNS.

**Conclusion/Significance:**

Taken together, these data suggest that Fra activity is required to correctly regulate Abl-dependent cytoskeletal dynamics underlying commissure formation. In the absence of Fra, increased Abl activity appears to be incorrectly utilized downstream of other guidance receptors resulting in a loss of commissures and the abnormal projections of some axons beyond the CNS/PNS border.

## Introduction

Attractive and repulsive guidance cues originating at or near the midline help guide the formation of the Drosophila embryonic nervous system. Receptors for these cues initiate intracellular signaling pathways that govern axon outgrowth and steering as well as formation of dendritic branching patterns [1]–[4]. Commissures form as axons integrate information from chemoattractive Netrins guiding them towards the midline and Slit-dependent repulsion preventing them from crossing [5]–[9]. Ectopic misexpression studies indicate that these attractive and repulsive systems mostly work independently of each other [10], but there is evidence of a fine interplay between systems at the transcriptional level [11], [12] and downstream of receptors.

In Drosophila, Netrins are midline attractants detected by Frazzled, a receptor expressed on most CNS neurons and in its absence many posterior commissures fail to form [5], [6], [13]. Fra may also have a non-cell autonomous effect guiding selected neurons at the segmental boundary [14]. A second Netrin receptor, the Down's Syndrome Cell Adhesion Molecule, Dscam [15], is also expressed on most neurons, and mutations in *fra* and *Dscam* interact to further reduce commissure formation [16], [17]. Since some commissures still form in *Netrin* null embryos, the presence of an additional Netrin-independent attractive system has been proposed [9], [16]. This is supported by evidence that Dscam may respond to a non-Netrin cue [16] and the recent identification of *turtle* as an important cell adhesion molecule that interacts with mutations in *Netrin* or *fra* to further reduce commissure formation [18].

At the midline, Slit-mediated repulsion prevents ipsilateral axons from crossing the midline and commissural axons from re-crossing the midline once on the contralateral side [7], [8]. Slit is detected by Roundabout, a receptor expressed on most axons, and in its absence many ipsilateral axons meander across the midline; over-expression of Robo can also reduce commissure formation [10], [19], [20]. Two additional Robo-family members also help guide axons at the midline and/or position them within the longitudinal connective [21]–[24]. Genetic elimination of *commissureless* (*comm.*) prevents commissures from forming, and Comm appears to play a major role in attenuating Robo-dependent repulsion [20], [25], [26]. Comm is a membrane protein expressed in commissural neurons and its expression may be partially regulated by Fra as commissural axons first extend towards midline [12]. Comm is known to bind to Robo receptors to help clear it from the membrane via endocytosis and sorting pathways [27]–[30], although more recent data suggest that Comm may also initiate intracellular signaling pathways that attenuate midline repulsion independent of its ability to clear Robo from the membrane [31].

Like most cell surface receptors, both Robo and Fra use conserved motifs within their cytoplasmic domains to signal information to the cytoskeleton. Fra has three evolutionarily conserved P-motifs (P1, P2 and P3) in its cytoplasmic domain and it appears that P3 is particularly important for Fra signaling *in vivo* [32], [33]. Signaling pathways originating from these motifs are thought to govern key aspects of the cytoskeletal dynamics underlying axon extension and steering [33]–[37]. Similarly, Robo initiates a repulsive response via activation of several signaling pathways initiated from one of the four (CC0, CC1, CC2 and CC3) conserved domains [38]–[46]. Of particular interest here is the Abelson tyrosine kinase signaling pathway, as both Abl itself and some of its key substrates (Ena) have been implicated in both Robo and Fra signaling.

Abl is a multifunctional tyrosine kinase that serves to link membrane receptors to actin dynamics underlying cell movement [47], [48]. In Drosophila, zygotic loss-of-function mutants of *abl* display mild defects in the axon scaffold including ectopic midline crossing errors [40], [41], [49]. These crossing errors are enhanced if *robo* itself is decreased or if mutations in genes known to interact with Robo are introduced [38], [41], [44]. Crossover defects and/or fused commissures are also common phenotypes observed when *abl* mutants are combined with mutations in a variety of genes implicated in the regulation of cytoskeletal dynamics (e.g. [38], [39], [44], [50], [51]). The observation that Abl binds to and phosphorylates the cytoplasmic tail of Robo led to the suggestion that Abl is a key regulator of actin dynamics during the transduction of midline repulsive cues [41], [49]. However, other data suggest Abl's role is not confined to the transduction of midline repulsion. When the zygotic and maternal contribution of *abl* is eliminated, commissures are missing and gaps appear in the longitudinal connectives [52]. Increasing levels of Abl activity also interact with heterozygous *robo* mutants to induce ectopic crossovers, and overexpression of Abl in a *comm* mutant, experiencing high levels of midline repulsion, actually improves commissure formation [49]. This suggests that increasing Abl activity could enhance midline attraction.

Indeed, Abl has been linked to the transduction of midline attractive cues. In GST-pull down and immunoprecipitation assays, Abl binds to the cytoplasmic tail of Fra and, when Abl is expressed in S2 cells with Fra, the tyrosine phosphorylation levels of Fra increase [36]. *In vivo*, very few commissures form in *fra, abl* double mutants [36]. Interestingly, *abl* mutations also enhance the degree of commissure loss observed in combination with mutations in *Dscam* [16], *turtle* [18], *amalgam* and its receptor *neurotactin* [53], *fasciclin I* [54], and *midline fasciclin* [55]. Together, this suggests that Abl plays multiple roles at the midline as it aids in the transduction of both Netrin dependent and independent attractive cues. To further elucidate Abl's role, we sought to elevate Abl activity in embryos that are null for *fra*. It was reasoned that if Abl is working with Fra to potentiate attractive signaling and a loss of *abl* in a *fra* mutant enhances commissural defects, then over-expression of Abl in a *fra* mutant should partially rescue commissure formation. However, as described herein, we observed the opposite: *fra* commissural defects are dramatically enhanced as both anterior and posterior commissures fail to form and this is associated with a large number of CNS axons projecting beyond the CNS/PNS border. To investigate the mechanism by which Abl may be causing this paradoxical interaction with *fra*, we manipulate midline repulsive elements and other genes known to regulate cytoskeletal dynamics. Our results suggest that in order to respond correctly to midline attraction, Fra is required to regulate Abl activity, in part, to direct the cytoskeletal dynamics underlying movement across the midline. In the absence of Fra, Abl appears to perform these functions downstream of other guidance receptors to induce a variety of projection errors, including trajectories away from the CNS.

## Methods

### Stocks

Stocks were raised on conventional cornmeal and molasses based media at room temperature or 25°C. All of the stocks used in this study have been fully characterized in previous studies. The Fra deletion mutant transgenes (UAS-Fra^ΔP1^, UAS-Fra^ΔP2^, UAS-Fra^ΔP3^ and UAS-Fra^wt^) and the 1407-Gal4 driver on chromosome II were described [32], [37]. Other constructs were described as follows: Ablwt, AblKN and BcrAbl [49], constitutively active MLCK [56], Rac^V12^ and Cdc42^V12^ [39] and sema2b-*tau-*myc P-element (Bloomington Stock Center; [21], [22]). The phenotypes generated by these transgenes when expressed in wild-type embryos have been described in these reports. Here, all phenotypes are compared to a *fra* heterozygote with a 1407-Gal4 driver to control for the presence of this driver chromosome and emphasize the change that occurs in the homozygote. Any differences between wild type and a *fra* heterozygote are briefly described in the text. Conventional breeding strategies and/or recombination experiments are used to combine gene mutations (e.g., *fra^4^*), the Gal4 driver and/or UAS transgenes in the same fly. Stocks were confirmed by complementation tests and/or PCR screening for P-elements as described previously [32]. To identify the genotypes of stained embryos, β-galactosidase (β-gal) marked balancer chromosomes are present in all final stocks.

***Histology:*** Standard methods are used to collect eggs at 25°C and stain them with mAb BP102 or mAb 1D4 against Fasciclin II (FasII) and the HRP-linked goat anti-mouse secondary antibody. The genotype of the embryos was established by X-Gal staining that identified balancer chromosomes. Mutant phenotypes in the axon scaffold were scored and at least three replicates were analyzed using a Student's t-test. Loss of commissures and ectopic crossovers were assessed as previously described [37], while AEP defects were scored based on the presence of a BP102 stained axon beyond the CNS/PNS boundary. Normally BP102 stains only CNS axons, and while motor nerve routes within CNS neuropile may be lightly stained, they are usually devoid of staining once in the PNS. Thus, BP102 staining of axons beyond the CNS/PNS boundary is a readily quantifiable phenotype. To observe Sema2b-expressing commissural axons we introduced a sema2b-Tau*-*myc P-element (Bloomington stock center; [21], [22]) into the background of our *fra^3^* 1407-Gal4 driver line and used an antibody against the myc epitope to detect tau-myc expression.

### Expression constructs

HA epitope tagged Fra^wt^ and Fra^ΔC^ transgenes cloned into the metallothionine vector (pMT) were described previously [36]. Individual P-motifs were deleted as defined in Kolodziej et al. [13]. pMT-Fra^ΔP1^ is deleted for PPDLWIHHDQMELKNIDK; pMT-Fra^ΔP2^ is deleted for TIESSKRGHPLKSFSVPGPPPTGGATPVTKHTP; pMT-Fra^ΔP3^ is deleted for ELNQEMANLEGLMKDLSAITANEFEC. All three P-motif deletion constructs contain a C-terminal HA epitope tag. To make myc-tagged versions, we first modified a commercially available S2 cell metallothionine vector (pMT-V5His, Invitrogen) to include a hygromycin resistance cassette (herein pMT-Hygro), and then introduced a myc epitope tag and tobacco etch virus protease site (TEV) just before this vector's V5 epitope and His tags. Then, using the HA-tagged versions as templates, we PCR amplified Fra wild-type (Fra^wt^) and P-motif deletions (Fra^ΔP1^, Fra^ΔP2^, Fra^ΔP3^) and cloned them into unique SpeI and PvuI sites of this parental vector.

### S2 cell culture and Immunoprecipitation Studies

Drosophila S2 cells were maintained in Schneider's media supplemented with 10% fetal bovine serum (complete media) at 27°C as per the conditions in Drosophila Expression System (Invitrogen). Approximately 2×10^6^ cells were transfected (Effectene, Qiagen) with either myc- or HA-tagged Fra^WT^, Fra^ΔP1^, Fra^ΔP2^, and Fra^ΔP3^ using the procedure recommended by the manufacturer. For the HA-tagged version, selection was achieved by co-transfection with pCohygro. Stable lines were selected over several weeks using 300 µg/ml hygromycin (Invitrogen). Fra protein expression was induced with 700 µM copper sulfate, and, 24 hours later, Fra was activated by adding a mixture of Netrin A and Netrin B for 30 minutes at 27°C. The cells were harvested by centrifugation at 13000 rpm and lysed in NP-40 buffer [36]. Cell extracts were pre-cleared by incubating with goat anti-mouse IgG magnetic beads (Pierce) for 30 minutes at 4°C. Following a two-hour incubation with 1 µg mouse anti-HA antibody (Sigma), Frazzled-antibody complex was pulled down using the magnetic IgG beads. The bead-protein complex was boiled in 1X SDS sample buffer and loaded onto a 7.5% poly-acrylamide gel. Protein was transferred to PVDF membrane (Millipore) following standard procedures and probed with antibody. Fra pull down was confirmed by blotting against the epitope tag using rabbit anti-HA or anti-myc (Sigma) at 1∶10000. After stripping (50 mM Tris (pH 6.8), 2% SDS and 100 mM β-mercaptoethanol at 50°C for 30 minutes), membrane was re-probed with either rabbit anti-Drosophila Abl (kindly provided by David VanVactor), guinea pig anti-Drosophila Abl (kindly provided by Mark Peifer) or rabbit anti-Bcr (Cell Signaling) at 1∶1000 dilution. The amount of Fra pull down was confirmed using a rabbit anti-myc or HA antibody (Sigma). For co-transfections with Abl transgenes, stable lines expressing full length Fra or mutants were transiently co-transfected with pMT GAL4 and either UAS-Ablwt, UAS-AblKN, or UAS-BcrAbl (1∶20). Twenty-four hours later, the cells were induced for protein expression and complexes were isolated as described above. To examine tyrosine phosphorylation of Fra and its P-motif deletions, the above procedure was used except that Frazzled expressing cells were treated with pervanadate [36] before Netrin was added. Phosphotyrosine was detected using PY-20 (Upstate) at 1∶1500 and 1∶20000 goat anti-mouse-HRP (Jackson).

## Results

**Figure 1:**
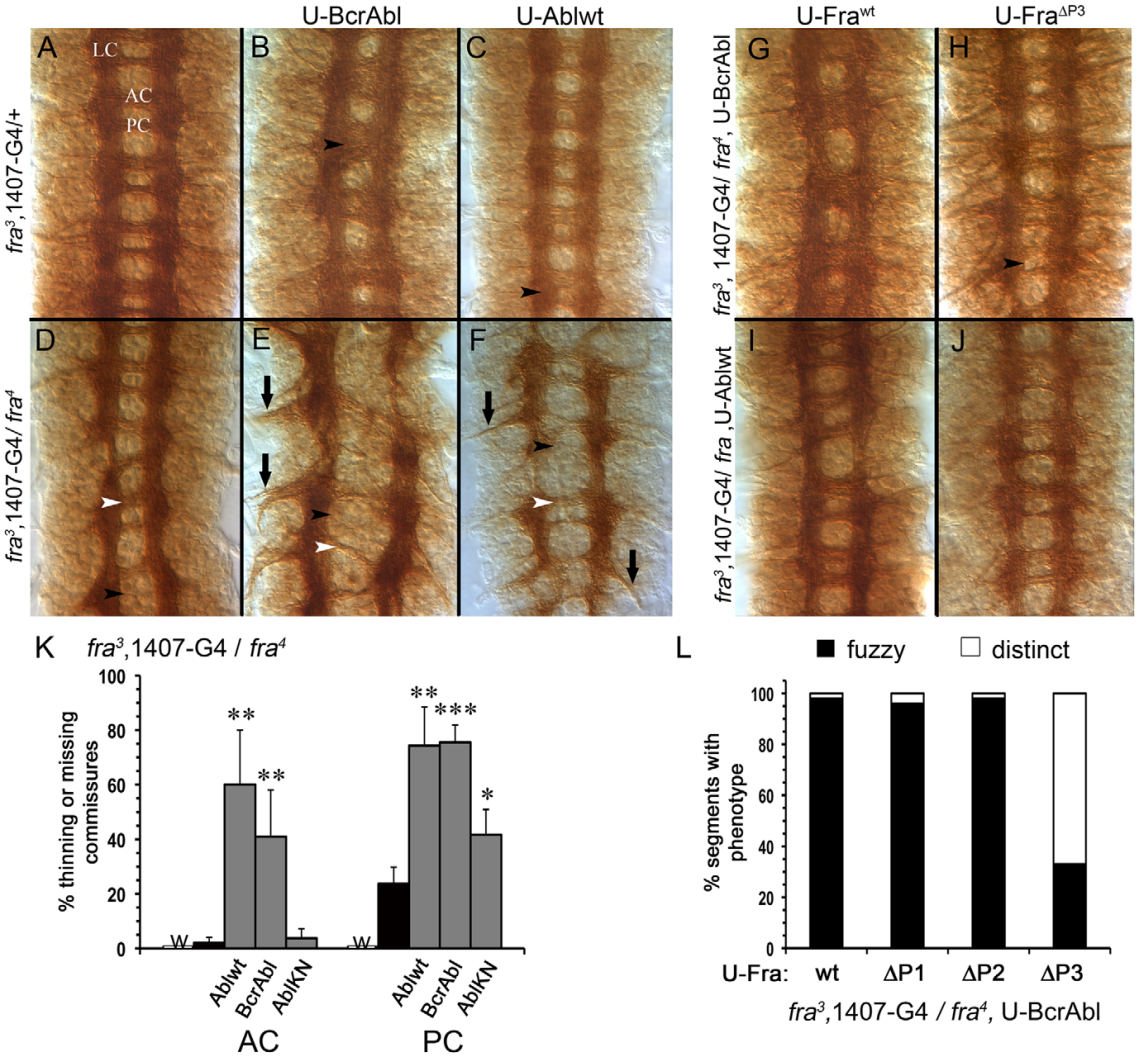
Increasing Abl activity in a *fra* mutant reduces commissure formation. Stage 16 embryos stained with mAb BP102 are depicted with anterior up. Indicated UAS- (U) transgenes are expressed pan-neurally using the *1407-Gal4* (1407-G4) driver line recombined onto a *fra^3^* chromosome. For this reason, phenotypes are compared to heterozygous *fra^3^* rather than a wild-type embryo; in most cases the heterozygote is similar to wild-type. [**A**] Normal anterior commissures (AC), posterior commissures (PC) and longitudinal connectives (LC) are observed in a *fra^3^ 1407-GAL4* heterozygote stained with BP102. [**B**] Over-expression of BcrAbl in a *fra^3^* heterozygote causes fuzzy commissures in most segments while [**C**] expression of Ablwt causes only a rare fuzzy commissure (black arrowheads). [**D**] Homozygous *fra^3^/fra^4^* mutants display thinning and missing PC's (black arrowhead) in some segments but the AC remains unaffected (white arrowhead). Over-expression of either [**E**] BcrAbl or [**F**] Ablwt in a *fra^3^/fra^4^* background enhances the degree of thinning and missing PC's (black arrowhead), and many AC's (white arrowhead) disappear. [**K**] Quantification of defects in the AC and PC are graphed with percent of commissures missing in a *fra^3^* 1407-Gal4 heterozygote (white, highlighted with ‘w’), *fra^3^* 1407-Gal4/*fra^4^* homozygote (black) and *fra^3^* 1407-Gal4/*fra^4^* homozygote's expressing the indicated Abl transgene (gray). All three Abl transgenes significantly enhance the loss of PCs and both BcrAbl and Ablwt affect AC formation (*P<0.05; **P<0.01; ***P<0.001). [**G**] Co-expression of a Fra^wt^ transgene restores the fuzzy commissure defects caused by expression of BcrAbl but [**H**] with the Fra^ΔP3^ transgene the AC and PC commissures are more distinct. Expression of [**I**] Fra^wt^ or [**J**] Fra^ΔP3^ transgenes also rescues commissure formation when Ablwt is over-expressed in a homozygous *fra* mutant. [**L**] The distribution of segments containing fuzzy (black) or distinct (white) commissures in *fra^3^/fra^4^* embryos co-expressing BcrAbl and each of the Fra P-motif deletion mutants is graphed.

Previous studies showed that loss-of-function mutations in *abl* enhance the degree of commissure loss in a *fra* mutant [36] while over-expression of a constitutively active form of Abl (Bcr^210^Abl, herein just BcrAbl) induces axons to ectopically cross the midline especially when wild-type Fra (Fra^wt^) is co-expressed [37]. Thus, it seems possible that Abl helps mediate midline attractive signaling, and if so, a simple prediction would be that over-expressing wild-type Abl, or BcrAbl in a *fra* homozygote would partially rescue *fra*-dependent defects in commissure formation. Accordingly, using the GAL4–UAS system and previously characterized stocks [32], [37], we over-expressed UAS-Ablwt (U-Ablwt) or UAS-BcrAbl (U-BcrAbl) in all neurons of a homozygous *fra* mutant (*fra^3^/fra^4^*) embryo and assessed commissure formation using the monoclonal antibody BP102. This antibody stains the anterior and posterior commissures (AC and PC, respectively) as well as longitudinal connectives (LC) running on either side of the midline [Figure 1A]. We were surprised to find that, rather than rescuing *fra^3^/fra^4^* commissural defects, elevating Abl activity actually enhanced the loss of commissures ([Figure 1E, F] black arrowhead, PC; white arrowhead, AC). Another striking defect was also observed: large bundles of axons exiting the CNS, often extending beyond the CNS/PNS boundary ([Figure 1E, F] arrows) (herein designated AEP, Axons Exiting to Periphery).

### Fra interacts with Abl to form commissures

One of the predominant defects in *fra^3^/fra^4^* mutants is thinning and missing posterior commissures, with nearly a quarter [24%] of segments showing this defect ([Figure 1D], black arrowhead; graphed in K; refs [13], [32], [36]). Over-expression of BcrAbl significantly (P<0.001) enhances these defects nearly three fold with 76% of segments having a thin or missing PC ([Figure 1E], black arrowhead; quantified in K). In addition, the AC, which is virtually unaffected in *fra^3^/fra^4^* homozygote, was thin or missing in 41% of segments (P<0.01; [Figure 1E], white arrowhead; graphed in K). This reduction in commissural axons crossing the midline was particularly surprising since expression of BcrAbl in a wild-type embryo results in fuzzy commissures, a defect caused by extra axons crossing the midline (see [37]). Fuzzy commissures remain evident in a *fra^3^* heterozygote ([Figure 1B], black arrowhead; see below). To test whether this result was unique to BcrAbl, we expressed wild-type Abl (U-Ablwt) in a *fra^3^/fra^4^* mutant background. In wild-type embryos, expression of Ablwt alone is innocuous [49], although a rare commissure may be malformed in a *fra^3^* heterozygote (see arrowhead [Figure 1C]). When Ablwt is expressed in a *fra^3^/fra^4^* mutant the phenotype is strikingly similar to that caused by expression of BcrAbl, with a three-fold (P<0.01) enhancement in PC defects and the emergence of AC defects ([Figure 1F], note that AEP defects are also present, black arrow; quantified in K). The enhancement in commissure defects partially requires Abl kinase activity. While pan-neural expression of a kinase dead version of Abl (U-AblKN) on its own has no phenotype in either wild-type or *fra* heterozygous embryos, expression in a *fra^3^/fra^4^* mutant still significantly (P<0.05) increases the frequency of thin and missing PC defects to about half that observed with over-expression of either Ablwt or BcrAbl; expression of AblKN spares the AC [Figure 1K]. Thus, both increasing and decreasing [36] Abl activity in the absence of Fra reduces the ability of commissural axons to cross the midline.

It is intriguing that such a striking phenotype is uncovered when Fra is removed since pan-neural expression of Ablwt on its own has very few defects and pan-neural expression of BcrAbl actually causes axons to erroneously cross the midline [49], [57]. This is also true when Abl signaling is increased in heterozygous *fra* mutants [Figure 1B, C]. This phenotype depends on the presence of Fra protein, as re-expression of Fra^wt^ in our mutants reverts the phenotype back to that seen when either BcrAbl or Ablwt is expressed alone: normal AC and PC formation is observed with Ablwt, and the fuzzy commissures seen with BcrAbl return [Figure 1G, I].

### A specific P-motif of Fra is not required to interact with Abl at commissures

In a previous gain-of-function assay, expression of BcrAbl in a subset of FasII expressing neurons induces ectopic crossovers, and these are enhanced by co-expression of Fra as long as the P3-motif is intact [37]. Therefore, we tested whether a specific P-motif is also required for Fra to revert the loss in commissural axon crossing. Essentially, we compared the ability of Fra^wt^ to revert the commissural defects with our previously described series of Fra mutants in which the individual P-motifs have been deleted (U-Fra^ΔP1^, U-Fra^ΔP2^ and U-Fra^ΔP3^; [37]). All of the Fra P-motif deletion mutants were able to significantly restore commissure formation in *fra^3^/fra^4^* mutants expressing Ablwt [Figure 1J, L]. In *fra^3^/fra^4^* mutants expressing BcrAbl, both Fra^ΔP1^ and Fra^ΔP2^ were able to revert the commissure loss seen in this mutant and in fact restore the fuzzy commissure defects similar to that observed with Fra^wt^ [Figure 1L]. While expression of U-Fra^ΔP3^ also restores commissure formation, the frequency of fuzzy commissures is significantly (P<0.001) reduced compared to Fra^wt^, as many segments now exhibit a distinct AC and PC ([Figure 1H], black arrowhead, [Figure 1L]).

### BcrAbl specifically interacts with the P3 motif to induce ectopic crossovers

**Figure 2:**
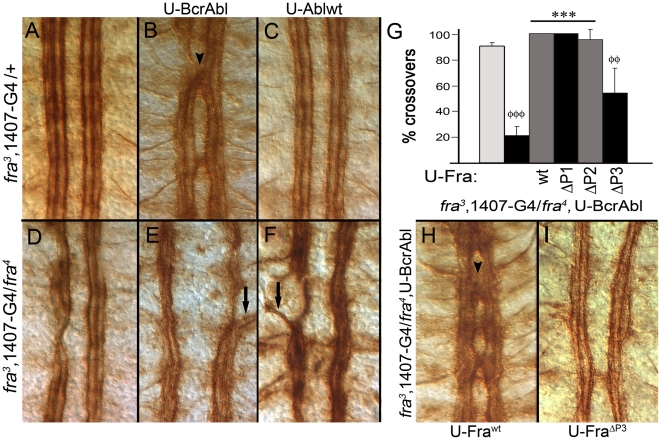
BcrAbl induced crossovers depend on the P3-motif of Fra. Stage 16 embryos stained with mAb 1D4 against Fasciclin II (FasII) are depicted with anterior up. All UAS (U) transgenes are pan-neurally expressed using the 1407-Gal4 (1407-G4) driver line recombined onto the *fra^3^* chromosome; thus, phenotypes are compared to heterozygous *fra^3^* rather than a wild-type embryo. [**A**] A *fra^3^* heterozygote exhibits a nerve cord that is essentially wild-type. Expression of [**B**] BcrAbl but not [**C**] Ablwt results in FasII axons crossing the midline incorrectly (arrowhead in B). [**D**] In homozygous *fra^3^/fra^4^* mutants, the FasII fascicles may partially fuse, and small breaks in the connective may appear. Removal of both copies of *fra* abolishes [**E**] BcrAbl and [**F**] Ablwt induced crossover defects. In both cases, FasII expressing axons are observed projecting towards the periphery (arrows), including axons from the medial most pCC/MP2 pathway (arrow in E). [**G**] Quantification of ectopic crossing errors (per embryo) observed using BcrAbl is graphed. White bar indicates crossovers in a *fra^3^* 1407-Gal4 heterozygote, black bar is a *fra^3^* 1407-Gal4/*fra^4^* homozygote and gray bars are *fra^3^* 1407-Gal4/*fra^4^* homozygote's expressing the indicated Fra transgene. Expression of BcrAbl in a *fra^3^* 1407-Gal4/*fra^4^* homozygote reduces ectopic crossovers, but these are restored by co-expression of Fra transgenes (* P<0.05, or ** P<0.01). Compared to Fra^wt^, expression of Fra^ΔP3^ restores only about half of the expected crossovers (φ P<0.05). [**H**] Re-expression of U-Fra^wt^ transgene restores the crossover defects (arrowhead in G) in *fra^3^/fra^4^* embryos expressing BcrAbl, while [**I**] expression of Fra^ΔP3^ restores about half of the ectopic crossovers.

The fuzzy commissure phenotype is often associated with extra axons ectopically crossing the midline between the AC and PC, a result known to occur with pan-neural expression of BcrAbl [49], [57]. Ectopic crossovers of FasII expressing neurons are readily observed using the monoclonal antibody 1D4. In wild-type embryos, FasII positive neurons extend axons in three parallel bundles on either side of the midline but never cross the midline [Figure 2A]. FasII fascicles tend to fuse together or form small gaps when both copies of *fra* are mutated [Figure 2D]. Consistent with a previous report [49], pan-neural expression of BcrAbl causes multiple FasII positive axons to ectopically cross the midline in most segments of nearly all embryos examined ([Figure 2B] arrowhead; [quantified in Figure2G]]. Removing both copies of *fra* significantly (P<0.001) reduces the frequency of these crossovers as only 21% of embryos still show a minor crossover or two ([Figure 2E]; quantified in [Figure 2G]). Confirming the BP102 results, these crossing defects depend on the presence of Fra protein as expressing Fra^wt^ with the 1407-Gal4 driver line restores the crossover defects [Figure 2G, H]. Expression of Fra^ΔP1^ or Fra^ΔP2^ also restores these crossing errors similar to Fra^wt^ [Figure 2G]. However, expression of Fra^ΔP3^ significantly (P<0.01) reduced the expressivity and penetrance of the crossing errors [Figure 2G, 2I]. Thus, the interaction between Fra and either Ablwt or BcrAbl during commissure formation does not depend on a specific P-motif. On the other hand, BcrAbl selectively interacts with the P3 motif of Fra to induce ectopic midline crossing errors. This is consistent with the reduction in fuzzy commissures noted with BP102 [Figure 1].

### Abl and BcrAbl bind to Fra independent of a specific P-motif

**Figure 3:**
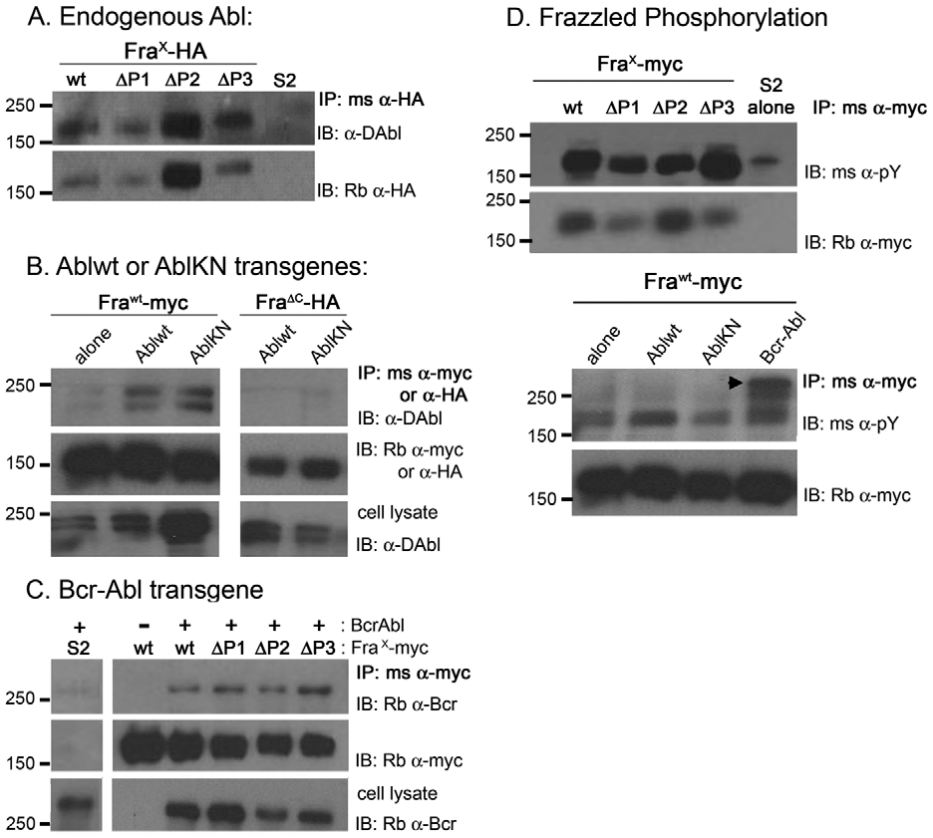
Both Ablwt and BcrAbl bind to Frazzled independently of a specific P-motif. [**A**] Stable Drosophila S2 cells were created that express an HA-tagged form of wild-type [wt] or P-motif deletion (ΔP1, ΔP2, ΔP3) mutants of Frazzled (Fra^x^-HA). Frazzled was immunoprecipitated (IP) from cells using a mouse monoclonal anti-HA, and western blots were probed (IB) with guinea pig anti-Drosophila Abl (D-Abl; top) or rabbit anti-HA (bottom). Compared to the amount of Frazzled receptor pulled down, endogenous Abl binds to Frazzled independently of a P-motif. [**B**] Transiently transfected Ablwt or AblKN (kinase inactive) genes also bind to a myc-tagged version of wild-type Fra (Fra^wt^-myc])but not to a HA-tagged receptor missing its cytoplasmic domain (Fra^ΔC^-HA). The amount of Fra (middle row) and Abl (bottom row) present in the lysates is indicated. [**C**] Activated BcrAbl, detected by a rabbit antibody against the Bcr region, also binds to Fra-myc even if one of the P-motifs is removed. No band is observed in mock IPs from S2 cells expressing only BcrAbl (far left). The amount of Fra pulled down (middle row), and the amount of BcrAbl available in lysates (bottom row) is shown. [**D**] In the top panel, S2 cells were pretreated for 30 min with pervanadate and then wild-type or P-motif deletions of Frazzled were immunoprecipitated using mouse anti-myc and the degree of tyrosine phosphorylation assessed by probing a blot with mouse anti-phosphotyrosine (α-pY; top row). Compared to the amount of receptor pulled down (bottom row), the level of tyrosine phosphorylation does not appear to be diminished when a P-motif is deleted. The bottom panel assesses Fra^wt^ phosphorylation when Abl transgenes are co-expressed in S2 cells that were not pretreated with pervanadate. Compared to the level observed in S2 expressing only Fra^wt^-myc, phosphotyrosine levels increase slightly if cells are co-transfected with Ablwt or BcrAbl, but not AblKN (bottom blot). In this blot, autophosphorylation of BcrAbl denoting its activated state is apparent (arrowhead). The position of molecular weight standards (in kDa) is indicated on the left side of each panel.

Our genetic rescue data suggest that Fra regulates Abl activity independent of a specific P-motif, while the P3 motif may have a special interaction with hyperactivated BcrAbl. To further address this, we asked whether these genetic interactions are reflected in a physical interaction between Abl and Fra. Forsthoefel et al. [36] noted that Abelson binds to the cytoplasmic tail of Frazzled but did not determine if the binding required a specific P-motif. Using Drosophila S2 cells, we created two sets of stable lines expressing either HA- or myc-tagged wild-type or P-motif deletion mutants of Fra under the control of a metallothionine promoter. When compared to the level of HA-tagged receptor pulled down in these immunoprecipitation assays, endogenous Abl continues to bind to Frazzled even when an individual P-motif (ΔP1, ΔP2 and ΔP3) is deleted [Figure 3A]. Similar results were observed using the myc-tagged versions of wild-type Fra and its P-motif deletions. When co-expressed in S2 cells, both wild-type (Ablwt) and kinase inactive (AblKN) transgenes bind to wild-type Fra as long as the cytoplasmic domain is present [Figure 3B], although it is noted that only a small fraction of the available protein is found in complex with each other.

Unlike wild-type Abl, BcrAbl specifically interacts with Fra to induce ectopic crossovers as long as the P3 motif is present. To ask if this genetic interaction is reflected in a specific binding of BcrAbl to the P3 motif of Fra, we transiently transfected S2 cell lines expressing myc-tagged versions of wild-type or P-motif deletion mutants of Fra with a BcrAbl transgene. Fra^wt^ or its P-motif deletions were immunoprecipitated using the C-terminal myc-tag and BcrAbl binding assessed using a commercial antibody against the Bcr component. Like wild-type Abl, BcrAbl clearly binds to Fra independently of a P-motif [Figure 3C]; thus, the selective genetic interaction between BcrAbl and the P3 motif of Fra is not reflected in differential binding properties.

Forsthoefel et al. [36] demonstrated that increased expression of Abl in S2 cells also led to increased phosphorylation of Fra, so we next asked if Fra phosphorylation is altered by removal of a P-motif. Fra or its P-motifs were immunoprecipitated from stable S2 cell lines using the C-terminal myc-tag and assessed for phosphotyrosine levels by western blot. Considering the amount of receptor precipitated, phosphorylation of Fra is largely unaffected by deletion of P1 or P2, while deletion of P3 may increase the signal ([Figure 3D], top). In the blot shown, cells were pretreated with pervanadate (∼30 min) to increase signal intensity and to demonstrate the lack of P-motif dependence. Phosphorylation is also observed in the absence of pervanadate treatment, although at a much lower level (see bottom panel [Figure 3D]). Phosphorylation of Fra^wt^ increases slightly if cells are transiently transfected with wild-type or BcrAbl transgenes, and it remains similar to S2 cells alone with AblKN, presumably reflecting the endogenous Abl activity. BcrAbl binding to Fra, as well as its hyperactivation, is confirmed in this blot by the presence of an autophosphorylated BcrAbl band at the top of the gel (arrowhead). Together, these immunoprecipitation assays indicate that both Abl and BcrAbl binding to Fra and the state of Fra phosphorylation are independent of any specific P-motif. This is consistent with the ability of the deletion mutants to rescue commissure formation when Abl or BcrAbl is over-expressed in a *fra* mutant.

### Identifying neurons that exit the CNS

Expression of Abl in a *fra* mutant also results in dozens of axons exiting toward the periphery (AEP defects), and, surprisingly, this defect is readily observed using BP102. Generally considered a marker of all CNS axons, BP102 clearly labels AC, PC and LC axons, and lightly stains ISN and SN nerve routes within the CNS, but it does not normally stain axons beyond the CNS/PNS boundary. Therefore, in mutant embryos, dense BP102 staining of axons beyond the CNS/PNS boundary clearly delineates a novel phenotype, herein termed an AEP defect (Axon Exiting to Periphery). Admittedly, no information on the identity of the exiting axons is provided with BP102, but this is addressed below.

**Figure 4:**
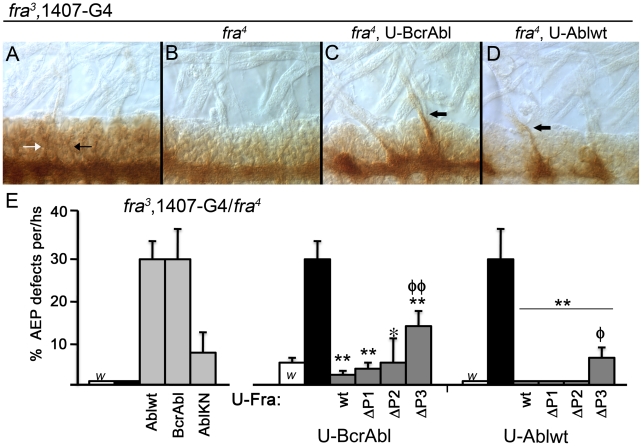
Quantification of axons exiting to the periphery [AEP] defects. A close up of the CNS/PNS border of stage 16 embryos stained with mAb BP102 is depicted with anterior to the right. Peripheral muscles are seen by Normarski optics [**A-D**]. UAS transgenes are expressed using the 1407-Gal4 (1407-G4) driver line recombined onto the *fra^3^* chromosome. [**A**] A *fra^3^* heterozygote is identical to a wild-type embryo and arrows indicate lightly stained ISN (black arrow in A) and SN (white arrow in A) nerve roots. Note the absence of BP102 staining beyond the CNS/PNS boundary. AEP defects are not observed in either [**A**] *fra^3^* heterozygote or [**B**] *fra^3^/fra^4^* homozygote embryos. Homozygous *fra^3^/fra^4^* embryos expressing either [**C**] BcrAbl or [**D**] Ablwt show large bundles of BP102 positive axons extending into the PNS (arrows in C, D). [**E**] Quantification of AEP defects per hemisegment (hs) is depicted for Abl transgenes (left) alone or in combination with Fra transgenes (right). White bars (highlighted with ‘w’) indicate *fra^3^* 1407-Gal4 heterozygote's, black bars are *fra^3^* 1407-Gal4/*fra^4^* homozygote's and gray bars are *fra^3^* 1407-Gal4/*fra^4^* homozygote's expressing the indicated transgene. All three Abl transgenes significantly (P<0.01) enhance AEP defects, although a kinase inactive Abl is less effective. BcrAbl (middle) and Ablwt (right) defects are significantly rescued by co-expression of any of the Fra transgenes, although rescue by Fra^ΔP3^ is significantly less than that observed using Fra^wt^. Comparison to *fra^3^* 1407-Gal4/*fra^4^* homozygote condition is indicated by *, while φ compares rescue of P-motif deletion to Fra^wt^ (*,φ P<0.05; **, φφ P<0.01).

Consistent with the known BP102 staining pattern, in wild-type and *fra^3^/fra^4^* mutant embryos we do not observe any AEP defects [Figure 4A, B & E]. When BcrAbl is expressed in a *fra* heterozygote, we observe minimal AEP defects and none in the same background with Ablwt [Figure 4E]. However, nearly a third of hemi-segments in embryos expressing BcrAbl or Ablwt in the absence of Fra exhibit AEP defects as many axons expressing the BP102 epitope actually extend past the CNS/PNS boundary ([Figure 4C & D], arrows). AEP defects are present in less than 10% of embryos over-expressing the kinase dead version of Abl (AblKN); this is significantly (P<0.01) less than either Ablwt or BcrAbl [Figure 4E]. Like the commissural axon defects, the AEP defects are rescued by re-expression of Fra^wt (^P<0.01), Fra^ΔP1^ (P<0.01) or Fra^ΔP2^ (P<0.05). A small (6%) but significant (P<0.01) frequency of AEPs is still observed if Fra^ΔP3^ is over-expressed [Figure 4E]. Therefore, Fra and Abl also interact to regulate which axons may leave the CNS.

**Figure 5:**
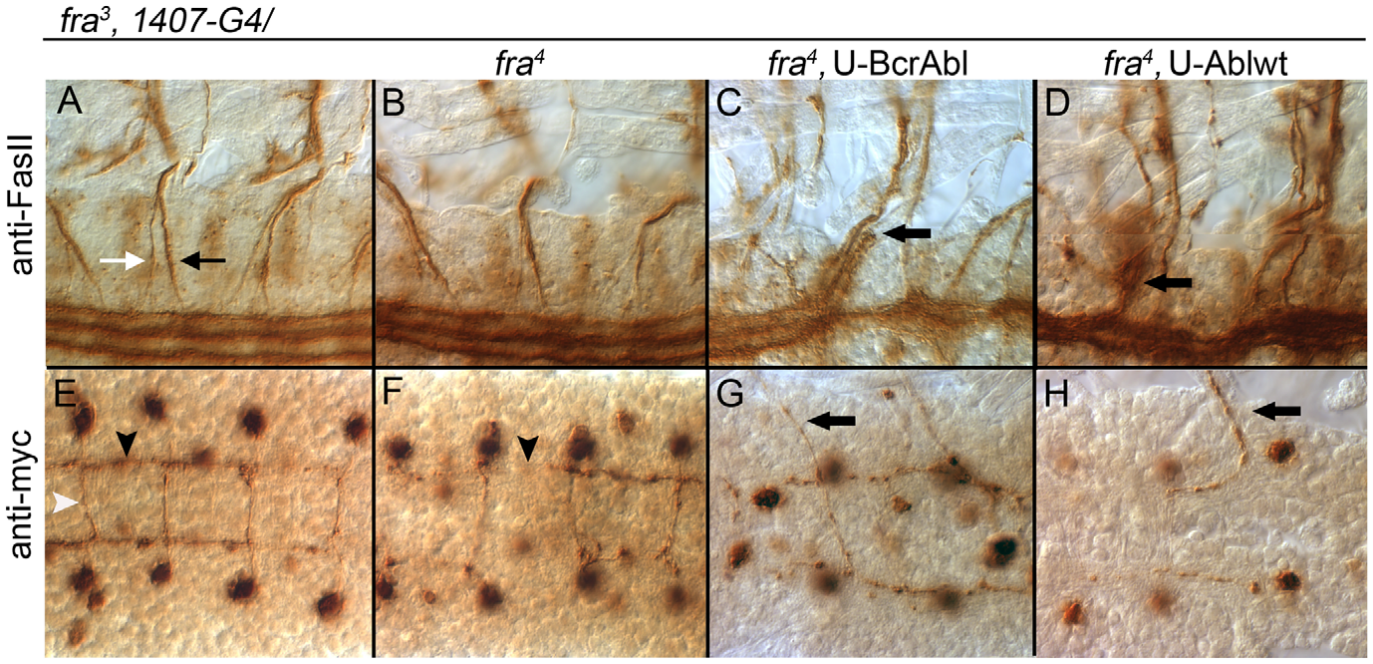
FasII positive longitudinal axons and *sema*2b expressing commissural axons leave the CNS in *fra* mutant embryos expressing BcrAbl or Ablwt. Stage 16 embryos were stained with either mAb 1D4 against *FasII* [**A-D**] or anti-myc to visualize axons expressing the *sema*2b-*tau*-myc reporter [**E-H**]. Anterior is to the right. With 1D4, ISN (black arrow in A) and SN (white arrow in A) motor nerve roots are observed exiting the CNS in both [**A**] *fra^3^,* 1407-Gal4 heterozygote and [**B**] *fra^3^, 1407-G4/fra^4^* homozygote embryos. Using the 1407-Gal4 (1407-G4) driver, expression of either [**C**] BcrAbl or [**D**] Ablwt in a *fra^3^/fra^4^* homozygote causes additional FasII expressing axons (arrows) to leave the CNS, including axons normally within the medial most pCC/MP2 pathway (arrow in C). [**E-H**] A *sema2b*-Tau-myc reporter was used to assess a subset of commissural axons. [**E**] In a *fra^3^* heterozygote, *sema*2b expressing neurons extend axons into the anterior commissure (white arrowhead) and then turn anteriorly to form part of the longitudinal connectives (black arrowhead). [**F**] In homozygous *fra^3^/fra^4^* mutants most axon projections into the anterior commissure are still intact; however, extensions in the longitudinal connectives may be disrupted (arrowhead). In *fra^3^/fra^4^* embryos expressing either [**G**] BcrAbl or [**H**] Ablwt the axons of *sema*2b expressing neurons are observed projecting towards the periphery (arrows).

In our analysis of the fuzzy commissure defects, we noted that many of the longitudinal axons expressing FasII also project past the CNS/PNS boundary ([Figure 2E, F]; arrows). 1D4 stains both longitudinal axons that remain in the CNS and motor neurons, notably the segmental (SN) and inter-segmental (ISN) nerve bundles that exit the CNS to innervate muscles in the periphery ([Figure 5A], arrowheads). While the projection of some motor neurons is known to require Fra [13], [58], the motor nerve roots within the CNS are mostly intact ([Figure 5B], arrowheads). However, expressing either BcrAbl or Ablwt in a *fra^3^/fra^4^* mutant causes FasII positive longitudinal axons that normally remain in the CNS to exit. This also includes axons from the medial pCC/MP2 fascicle, which pioneers the longitudinal connective and normally does not exit the CNS ([Figure 5C], arrow; [59]). The fact that this fascicle is observed exiting the CNS is particularly interesting since the medial pCC/MP2 is normally the least affected in a *fra* mutant [32]. It is also clear that while many of the AEP defects track with the motor neurons forming the SN and ISN nerve roots, others exit with seemingly no association ([Figure 5C, D], arrows). Because we cannot readily distinguish motor neurons from other *FasII* positive axons we did not attempt to quantify the AEP phenotype using 1D4.

Given the reduction in commissures in *fra^3^/fra^4^* mutant embryos expressing either Ablwt or BcrAbl, we made use of the reporter construct, *sema*2b-*tau*-myc, to specifically examine the axon projections of a small subset of commissural axons. This reporter labels the axons of a subset of neurons in abdominal segments five through eight that project axons into the anterior commissure and then turn anteriorly to form a part of the media-lateral longitudinal connective [Figure 5E] [21]. In *fra^3^/fra^4^* mutants, most s*ema2b* axons still cross in the anterior commissure, consistent with this being the least affected commissure in *fra* mutants, and, while small breaks in the longitudinal projections can be observed, no axons leave the CNS ([Figure 5F], arrowhead). However, when Fra is absent, expressing BcrAbl or Ablwt causes these axons to project out of the CNS and into the periphery [Figure 5G, H]. In both of these mutants, *sema2b* axons exit at multiple points in their trajectory; some cross the midline and then exit, others exit while extending longitudinally, and still others do not cross the midline but, rather, extend directly away from the midline [Figure 5G, H]. Thus, increased Abl activity appears to affect guidance decisions at several choice points, and, surprisingly, some of these are severe enough to drive these axons out of the CNS.

### Decreasing midline repulsion does not rescue guidance defects

**Figure 6:**
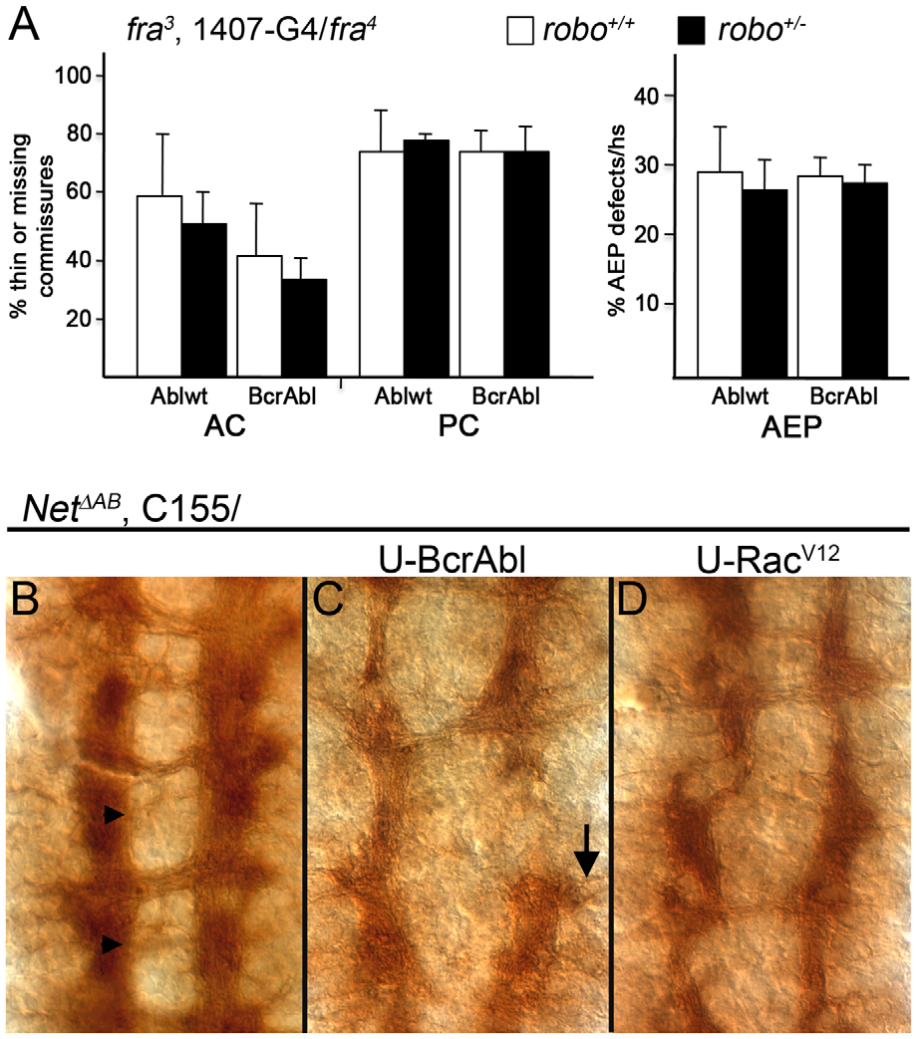
Changes in midline repulsive activity do not account for the Abl gain-of-function phenotypes. The ability of a heterozygous loss of *robo* to alter the phenotypes that occur when the 1407-Gal4 (1407-G4) driver was used to pan-neurally express Ablwt or BcrAbl in *fra* mutants was quantified after staining embryos with mAb BP102 (as described in [Figures 1 or 4]). [**A**] Graphed are the percentage of embryos with missing or thin anterior (AC) and posterior (PC) commissures as well as the percentage of hemisegments (hs) exhibiting axons exiting towards the periphery (AEP) in *fra^3^, 1407-G4/fra^4^* homozygote embryos (white; *robo^+/+^*) versus *fra^3^, 1407-G4/fra^4^* homozygote embryos carrying one copy of a null *robo* allele (black; *robo^+/−^*). The loss of one copy of *robo* has no significant affect on the frequency of these defects. [**B-D**] To test whether *netrin*-dependent repulsion is important, BcrAbl or Rac^V12^ (as indicated at top) were over-expressed in *netrin* null embryos (*net^ΔAB^*) using the pan-neural C155 Gal4 driver also on the X chromosome. [**B**] In a relatively severe *netrin* mutant, most posterior commissures (black arrowheads) are absent and several anterior commissures are thin. Most commissures are absent when either [**C**] BcrAbl or [**D**] Rac^V12^ are over-expressed in all neurons, and, with BcrAbl, some axon bundles also project beyond the CNS/PNS boundary (arrow in C).

Both too much and too little Abl causes ectopic midline crossing errors when Robo-dependent repulsion is reduced [42], [49], and Fra activity may regulate the level of Commissureless, which in turn governs Robo surface accumulation (Yang et al., 2009). Thus, elevating Abl activity in a *fra* mutant might have shifted the balance of attractive and repulsive signals at the midline to reduce commissure formation and/or allow axons to leave the CNS. To test this idea, we introduced one copy of a *robo^1^* allele (effectively reducing Robo1 dependent repulsion by half) into our *fra* mutants expressing either BcrAbl or Ablwt. Surprisingly, decreasing Robo-dependent repulsion had no observable effect on either of the phenotypes induced by Abl activity [Figure 6]. In a *fra* homozygote expressing Ablwt, thinning or missing AC's were seen in 60% of segments and 74% of segments had defects in the PC. Introduction of a *robo^1^* allele did not significantly change these numbers as commissures were thinning or missing in 50% of AC's and 78% of PC's [Figure 6]. We also observed no detectable difference in the AEP defects in these embryos: 29% of hemisegments in *fra^3^/fra^4^* embryos expressing Ablwt had this defect compared to 26% in a *robo^1^* heterozygote background [Figure 6]. A similar trend was seen with expression of BcrAbl in these mutant backgrounds [Figure 6]. This data suggests that an increase in Robo1-dependent repulsion is not likely to be the reason axons fail to cross at commissures or leave the CNS when Abl activity is increased in the absence of Fra.

In some cases, when Netrin is detected by Unc5 it can elicit a repulsive response [60]. Thus, we sought to test whether elevated Abl activity might be enhancing the repulsive activity of midline Netrins. To do this, the C155-Gal4 insertion was recombined onto the *Net^ΔAB^* mutant chromosome, which is null for both Netrin A and B genes [61]. Using this chromosome, BcrAbl was pan-neurally expressed in embryos that do not express either Netrin, but this still results in a loss of commissures and many axons exiting from the CNS [Figure 6B] similar to that observed in homozygous *fra* mutants. Thus, the Abl gain-of-function phenotypes do not appear to be the result of Netrin dependent repulsion.

### Fra regulates the cytoskeletal dynamics during commissure formation

Besides its interactions with axon guidance receptors at the midline, Abl is a key regulator of the cytoskeletal dynamics underlying growth cone advance and steering [47], [48]. Does increasing Abl activity alter the dynamics of this response and cause guidance defects? To test this idea, we sought to disrupt cytoskeletal dynamics in a *fra* mutant independent of Abl, but using gene mutations known to affect midline guidance.

**Figure 7:**
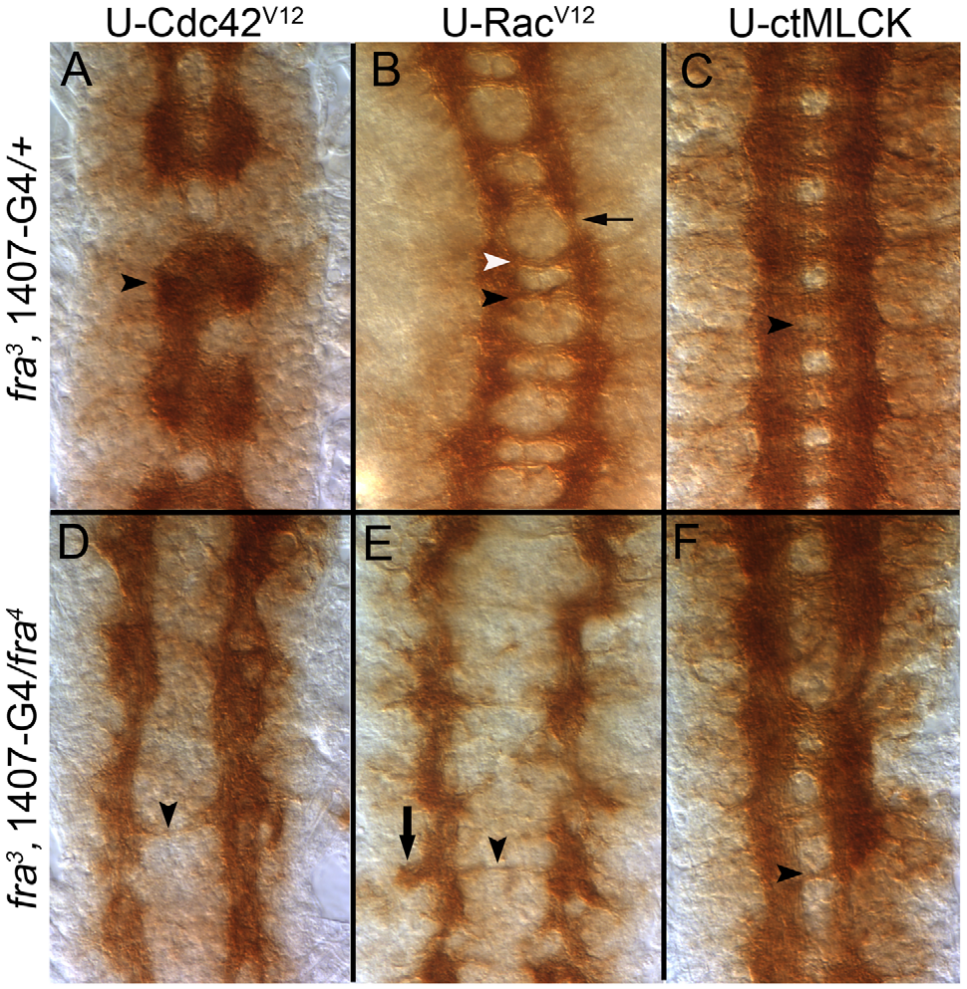
Expression of Rac^V12^ and Cdc42^V12^ reduces commissure formation in a *fra* mutant. Stage 16 embryos stained with the mAb BP102 are depicted with anterior up. UAS (U) transgenes are expressed pan-neurally using the *1407-GAL4* (1407-G4) driver line recombined onto the *fra^3^* chromosome, and, as in other figures, phenotypes are compared to heterozygous *fra^3^*. Phenotypes in heterozygous *fra^3^* embryos tend to be somewhat milder than that observed in a wild-type embryo (see text). [**A**] Expression of Cdc42^V12^ in a *fra^3^* heterozygote background results in fused commissures [arrowhead] and large gaps in the longitudinal connectives. [**B**] Expression of Rac^V12^ in a *fra^3^* heterozygote causes a thinning of both PC (black arrowhead) and AC (white arrowhead) as well as LC (black arrow). [**C**] Expression of ctMLCK in a *fra^3^* heterozygote results in fuzzy commissures (black arrowhead**)**. Expression of both [**D**] Cdc42^V12^ and [**E**] Rac^V12^ in a *fra^3^/fra^4^* mutant significantly reduces commissure formation (black arrowheads) and, with expression of Rac^V12^, some axons may orientate towards the periphery (arrow in E), but they are not observed to exit the CNS. [**F**] Expression of ctMLCK in a *fra^3^/fra^4^* mutant does not significantly alter the PC (arrowhead) and LC defects normally observed in a *fra* mutant.

Cdc42 regulates multiple elements of the cytoskeleton, and, in Drosophila, expression of a constitutively active form of the protein (U-Cdc42^V12^) in a specific subset of FasII positive neurons leads to ectopic crossovers, the frequency of which are halved by a heterozygous *fra* mutation [37], [39]. Like that observed in wild-type embryos, pan-neural expression of Cdc42^V12^ in a *fra^3^* heterozygote also causes severe errors in axon pathway formation as commissures fuse and large gaps appear in the LC ([Figure 7A], arrowhead). Yet, quite surprisingly, when Cdc42^V12^ is expressed in a *fra^3^/fra^4^* homozygote most commissures are absent in nearly every segment, and LC formation improves as fewer gaps are now observed [Figure 7A, D]. It is noted, however, that while over-expression of Cdc42^V12^ in a *fra^3^/fra^4^* mutant caused a dramatic loss in commissures, even more severe than over-expression of BcrAbl or Ablwt, no axons were observed leaving the CNS. These data confirm the importance of Fra signaling during formation of commissures and point to a key role for Abl in regulating cytoskeletal elements.

The severity of these Cdc42^V12^ phenotypes prompted us to test another protein, Rac, which also regulates actin dynamics and a constitutively active form of Rac (Rac^V12^) also causes FasII expressing axons to ectopically cross the midline [37], [39]. When Rac^V12^ is pan-neurally expressed in wild-type embryos or a *fra^3^* heterozygote [Figure 7B], commissures and longitudinal connectives appear thin and slightly disorganized. But when Rac^V12^ is over-expressed in a homozygous *fra* embryo, it too yields a major reduction in commissure formation [Figure 7E]. While some axons appear to orient away from the midline ([Figure 7E}, arrow), they were never observed to cross the CNS/PNS boundary.

Next, we sought to selectively elevate myosin activity in a *fra* mutant using a constitutively active Myosin Light Chain Kinase (U-ctMLCK) that increases myosin activity by phosphorylating the Regulatory Light Chain of myosin II [56]. Expression of ctMLCK in a subset of FasII positive axons induces ectopic midline crossing errors, which, like Rac^V12^ and Cdc42^V12^, are sensitive to heterozygous removal of *fra* [37]. Pan-neural expression of ctMLCK in wild-type embryos leads to ectopic crossovers (i.e. fuzzy commissures) in almost every segment leading [56]. While the frequency of this defect is somewhat diminished in a *fra* heterozygote, many segments still display fuzzy commissures ([Figure 7C], arrow). Unlike Cdc42 or Rac, however, expression of ctMLCK in *fra* homozygous embryos does not reduce commissure formation beyond that normally observed in a *fra* mutant [Figure 7F]. Similar results were observed for all three transgenes (U-Cdc42^V12^, U-Rac^V12^ and U-ctMLCK) when the pan-neural *elav*-Gal4 driver line was utilized (data not shown). Over-expression of Rac^V12^ in a *Netrin* null embryo (*Net^ΔAB^*) also reduces commissure formation [Figure 6B]; Cdc42 could not be examined with *Netrin* due to stock health.

Together, the Rac and Cdc42 data suggest that, in the absence of Fra, unilateral perturbation of actin cytoskeletal dynamics is sufficient to reduce commissure formation. These data continue to highlight the critical role Fra plays in regulating cytoskeletal dynamics during guidance events at the midline, and implicate all three molecules, Abl, Rac and Cdc42, in this process. Moreover, because Rac^V12^ and Cdc42^V12^ do not cause AEP defects, these data also suggest that Abl contributes unique information governing which axons may leave the CNS.

## Discussion

Frazzled and Abelson Tyrosine kinase activity clearly cooperate during the formation of embryonic commissures. In the absence of Fra, detection of Netrin-dependent chemoattraction is compromised and many posterior commissures fail to form. Both anterior and posterior commissures are absent if *fra* and *abl* activity is lost. This presumably reflects the ability of *abl* mutations to interact with a second Netrin receptor, Dscam, as well as Netrin independent receptors (e.g. Turtle) known to be important for commissure formation [16], [18]. Finally, as most commissures are also lost when both maternal and zygotic contributions of Abl are genetically removed, it seems Abl itself is required for commissure formation [52]. Given these different observations, it seemed plausible that over-expressing Abl in *fra* null embryos would improve commissure formation. However, instead of an improvement, we clearly document a major decrease in both anterior and posterior commissures and the induction of a novel phenotype whereby axons normally confined to the CNS now project into the periphery (AEP defects). It is worth emphasizing that these phenotypes occur even with the over-expression of a wild-type Abl transgene that retains its autoinhibitory domain and must be activated by endogenous mechanisms [47], [49]. These phenotypes are completely dependent on the absence of Fra but not any specific P-motif, occur in the absence of Netrins as well, and are not alleviated if Robo-dependent midline repulsion is reduced. Interestingly, the loss of commissures, but not the AEP defects, is also observed when activated Rac or Cdc42 GTPases are over-expressed in a homozygous *fra* mutant. Taken together, we propose that during exploration of the midline, Fra is a key regulator of Abl activity and helps determine how the cytoskeletal machinery utilizes Abl. In the absence of Fra, axon outgrowth does not simply stall; but rather, axons follow a variety of aberrant trajectories away from the midline. This suggests that Fra normally competes with several other receptor systems to dictate how Abl functions to regulate the cytoskeletal machinery. While competitors undoubtedly include other midline guidance cues, the emergence of AEP defects suggests that Fra also competes with guidance systems not normally associated with the midline. In the absence of Fra, these other receptors appear to utilize the extra Abl to alter cytoskeletal dynamics at a variety of choice points, ultimately preventing commissure formation and directing some axons out of the CNS.

The Abl gain-of-function phenotype described herein occurs only if *fra* is absent. That is, commissures form correctly in a heterozygous *fra* mutant or when partially active Fra transgenes with a single P-motif deleted are re-expressed with Abl. In S2 cell immunoprecipitation experiments, both Abl and BcrAbl bind to the cytoplasmic tail of Fra independent of any specific P-motif (see also [36]). While surprising given the conservation of these P-motifs and their known importance to Fra function [32], [33], the lack of P-motif specificity is consistent with our genetic rescue experiments. All three P-motif deletion mutants rescue commissure formation and the AEP defects elicited by over-expression of either wild-type Abl or BcrAbl in *fra* embryos. The ectopic midline crossovers (fuzzy commissures) observed with only BcrAbl also depend on Fra and specifically the P3-motif, confirming a previous report [37]. However, BcrAbl is not an endogenous Drosophila protein and, as discussed by others [62], the human Bcr domain may induce neomorphic phenotypes. Because BcrAbl does not preferentially bind to the P3 motif, and wild-type Abl does not elicit crossover defects, we now suspect that the ectopic crossovers are an example of a neomorphic phenotype, a hypothesis that will be extensively addressed in future work.

The physical interaction between Fra and either Ablwt or BcrAbl in immunoprecipitation assays can reflect direct or indirect association between the two proteins. It is possible that our failure to observe P-motif dependence reflects the binding of Abl to multiple P-motifs or the use of scaffold proteins associated with more then one P-motif. Given that Forsthoefel et al. [36] demonstrated that the cytoplasmic tail of Fra fused to glutathionine-S-transferase (GST) binds to *in vitro* translated Abl, direct binding of Abl to Fra is clearly possible. If so, these experiments suggest that Abl may bind to Fra in the regions between P-motifs, which is, in fact, where most of the tyrosine residues within the cytoplasmic domain of Fra reside [13], [36]. Moreover, in S2 cells, tyrosine phosphorylation of Fra is not affected by removal of the P1 or P2 motif and may actually increase when P3 is removed. This is intriguing as removal of the P3-motif is known to significantly affect Fra signaling *in vivo* [32], [33] and the Fra^ΔP3^ transgene is the least capable of rescuing the AEP defects caused by Ablwt or BcrAbl expression. Immunoblots of Fra phosphorylation in the absence of pervanadate pretreatment also suggest the steady-state level of tyrosine phosphorylation may be relatively low, or highly dynamic. S2 cells are known to express tyrosine phosphatases that antagonize Abl activity for some substrates [63], [64], and antagonistic action between Abl and several phosphatases during nerve cord development has been documented [50], [51], [65]–[67]. Since tyrosine phosphorylation of vertebrate DCC is required for attractive responses, axon outgrowth and orientation of the axon (e.g. [68]–[74]), it will be important to systematically assess how Fra and Abl physically interact to regulate each other's activity during midline guidance.

Both of the phenotypes observed when Abl activity is elevated in a *fra* mutant, the loss of commissures and the exiting of CNS axons to the periphery, suggest these embryos are experiencing an excess of midline repulsion. During commissure formation, Slit dependent repulsion prevents commissural axons from crossing unless Commissureless prevents the Slit receptor Robo from accumulating on the cell surface [9], [75]. Before commissural axons extend towards the midline, Fra activity may help increase Comm expression [12] so Comm levels are expected to be reduced in *fra* mutants leading to an increase in Robo-dependent repulsion. Since increasing Robo activity in a *fra* embryo is sufficient to reduce commissure formation [10], it was important to test whether an excess of Robo-dependent repulsion underlies the Abl over-expression phenotypes. However, our introduction of one null allele of *robo* (Robo1) had no discernable affect on the Abl gain-of-function phenotypes, even though, in previous work, elevating Abl activity in a heterozygous *robo* mutant induces ectopic midline crossing errors [49]. Given the absence of even a minimal suppression, it seems unlikely that the loss of commissures and/or AEP defects noted in our mutants reflects an increase in Robo-dependent midline repulsion. While two other Robo receptors, Robo2 and Robo3, operate during midline guidance [21]–[24], [75] and could conceivably contribute to these Abl phenotypes, neither of these receptors have the conserved CC3 cytoplasmic domain known to be important for Abl binding to Robo1 [45], [75]. Moreover, while certain Netrin receptors (e.g. Unc5; [60]) can also elicit a repulsive response, both commissure loss and AEP defects still occur when Abl is over-expressed in a *Netrin* mutant. This provides strong evidence that abnormal signaling by other Netrin receptors is not responsible for these phenotypes.

In terms of the AEP defects, which also point to excess repulsion, it is worth noting that axons do not leave the CNS in a *commissureless* mutant experiencing very high levels of Slit-dependent repulsion [20], [25], [49], nor do they appear evident in published figures of *fra Dscam* double mutants, or even *fra Dscam abl* triple mutants [16]. While the identity of all the axons leaving the CNS has not been established, we have confirmed that at least two subtypes of CNS axons are exiting: both FasII expressing interneurons and *sema2b* commissural axons. FasII axons do not leave the CNS when midline repulsion is elevated in a *commissureless* mutant [20], [25], [49], and while the level of Abl activity affects the trajectory of FasII interneurons, in most cases altering Abl activity leads to midline crossing errors rather than an exit from the CNS [42], [49], [51]. Over-expressing Abl in a *fra* mutant also affects several different guidance decisions by Sema2b-expressing commissural axons. While the cues guiding these neurons are not well understood, the spectrum of defects observed both before and after they cross the midline suggest that these neurons are responding to more then just midline repulsion. Thus, if a repulsive mechanism is functioning, as initially suggested by the phenotype, the origin of the signal remains to be determined. Indeed, our data using activated forms of Rac and Cdc42 suggest that the primary defect lies in the ability of growth cones to properly regulate actin dynamics underlying axon outgrowth and steering. This could involve both attractive and repulsive systems.

Proper axon guidance also requires concerted regulation of the cytoskeletal dynamics underlying axon outgrowth and steering [76]. Like most guidance receptors, Fra, or its vertebrate and C. elegans homologues, is known to initiate signaling pathways affecting cytoskeletal dynamics [34], [37], [77], [78]. Abl is also a key regulator of actin dynamics in vertebrate cells [47], [48], [79]–[81] and of the development of the *Drosophila* nervous system. Mutations in *abl* interact with several cytoskeletal regulators to affect axon pathway formation: *kette* [82], *capulet* [40], *chicadee* (Profilin; [51]), *enabled* [42], [83] and *trio* [36], [84], [85]. Thus, in the absence of Fra-dependent regulation, does elevated Abl activity affect the cytoskeletal machinery to indirectly cause a reduction in commissures and AEP defects? Here, we tested this basic concept by expressing in *fra* mutants other key regulators of cytoskeletal dynamics known to affect midline guidance [37], [39], [56]. Surprisingly, over-expression of activated Rac and Cdc42 in a *fra* mutant replicates the loss of commissure phenotype (but not the AEP defects) observed with Abl. The Cdc42 result is most intriguing as expression in a wild-type or heterozygous *fra* embryo results in fused commissures and gaps in the longitudinal connectives. Yet, upon complete removal of Fra, commissures do not form and the longitudinal connectives reform. Thus, in the absence of Fra, commissure formation, but not AEP defects, appears to be particularly sensitive to manipulation of actin-based processes. It is possible that our manipulation of Cdc42 and Rac activity in a *fra* mutant is affecting shared processes related to actin polymerization. For example, in vertebrate studies, Cdc42 and Abl work in parallel to regulate actin polymerization (e.g. [86]–[89]) and Abl may activate Rac in response to cell adhesion [87], [90], [91]. If so, our data suggest that in the absence of Fra activity these key regulators are being used by other surface receptors to regulate actin dynamics in a manner that ultimately prevents commissure formation. This is certainly consistent with the number of guidance systems that have been linked to these regulators [35], [92]–[94] and the scope of guidance defects detected in our embryos. Minimally, the Cdc42 and Rac data continue to highlight the degree and importance of Fra-dependent regulation of cytoskeletal dynamics, especially actin-based processes, during commissure formation. Moreover, they point to a highly competitive process between multiple surface receptors and the cytoskeletal machinery where Fra is a major player. While competition between midline attractive and repulsive cues has been recognized [2], [10], in our experiments, midline repulsive activity had no affect on the Abl phenotypes. Therefore, it seems likely that Fra is competing with several other receptor systems whose presence (but not identity) has been uncovered by over-expression of Abl, Rac or Cdc42 in homozygous *fra* embryos. Which guidance events are being affected has not yet been determined, but a few candidates exist. In addition to *fra* and *Dscam* [16], [36], loss-of-function mutations in *abl* interact with the cell-cell adhesion molecules *neurotactin* and *amalgam* [53], *fasI* [54], *midline-fasciclin* [55] and *turtle* [18] to reduce commissure formation and some of these are fairly ubiquitously expressed in the nerve cord. Abl has also been linked to the regulation of cell-cell adhesion molecules alone or in combination with receptor systems such as Notch [94], [95].

In summary, our data suggest a model whereby Fra activity initiates key signaling events that dictate when and how Abl activity is utilized during commissure formation. Rac and Cdc42 are probably also involved in this process, and, together with Abl, help regulate key aspects of actin dynamics underlying commissure formation. In the absence of Fra other midline guidance systems are still functioning well enough to form most commissures, but they are clearly sensitive to perturbation of intracellular signaling pathways regulating cytoskeletal dynamics. Thus, when Fra is removed, other guidance systems appear to recruit Abl, Rac or Cdc42 activity to misdirect axon outgrowth, ultimately preventing commissure formation and, with Abl, causing some axons to exit the CNS. Thus, in a normal embryo, Fra must be sending information that allows it to compete very well against these other guidance receptors to properly regulate axon outgrowth and steering during commissure formation. While an alteration in midline guidance decisions may also account for the AEP defects, the scope of guidance errors observed in neurons leaving the CNS suggest that increasing Abl activity could also be affecting other guidance systems not directly associated with the midline. While the identity and specific role of these guidance systems awaits discovery, the sensitivity of the CNS axon scaffold to Abl over-expression will be an important tool for identifying these competing pathways.
